# Cognitive, functional, and social disparities in patients receiving dialysis: a multi-site survey

**DOI:** 10.3389/frhs.2025.1688966

**Published:** 2026-01-20

**Authors:** Victoria Liou-Johnson, Aditya Narayan, Brandon E. Johnson, Nirav R. Shah, Unini Odama

**Affiliations:** 1 Clinical Excellence Research Center, Stanford University, Palo Alto, CA, United States; 2 Psychology Department, Palo Alto University, Palo Alto, CA, United States; 3 Clinical Psychology Department, Notre Dame de Namur University, Belmont, CA, United States; 4 Stanford University School of Medicine, Palo Alto, CA, United States; 5 Department of Cell and Molecular Biology, John A. Burns School of Medicine, University of Hawai’i, Honolulu, HI, United States; 6 Center for Bioethics, Harvard Medical School, Boston, MA, United States

**Keywords:** cognitive dysfunction, end stage kidney disease (ESKD), functional mobility, health equity, kidney care, patient centered care, social needs

## Abstract

**Introduction:**

End-stage kidney disease (ESKD) affects many Americans, with higher risks in certain subgroups of the US population. Differential kidney health outcomes may stem from *non-medical* social drivers of health, cognitive difficulties, and functional limitations. Recommendations for individuals with ESKD are often standardized and may not account for unique challenges and access barriers that individuals face. These challenges lead to preventable differences in access to treatments such as home dialysis and kidney transplantation. This study examines the prevalence of unmet social, cognitive, and functional needs amongst patients receiving dialysis and evaluates the intersection of these barriers to inform strategies to improve kidney health outcomes for all patients.

**Methods:**

In a cross-sectional study, a convenience sample of 962 patients from diverse backgrounds, currently undergoing dialysis from multiple dialysis centers across the United States (aged 21–95 years), were surveyed. Descriptive, Spearman's correlation, logistic regression, and Chi-Square Test analyses conducted.

**Results:**

From our large sample, 45.1% reported memory challenges, 19.6% required assistance with activities of daily living (ADLs), and 51.0% experienced two or more mobility limitations. Additionally, 20.4% reported difficulty accessing healthcare, while 16.3% faced challenges obtaining medications. A subset (12.2%) of participants experienced overlapping social, cognitive, and functional barriers. Unmet needs were disproportionately higher amongst public insurance participants compared to those with private insurance, with 33.0% of Dual-eligible participants reporting three or more unmet needs.

**Discussion:**

This study highlights the significant intersection of social, cognitive, and functional barriers faced by patients receiving dialysis with ESKD, particularly those from vulnerable populations. Addressing these multifaceted needs through person-centered interdisciplinary care models and policy interventions is critical to reducing disparities and improving outcomes in kidney health outcomes.

## Introduction

End-stage kidney disease (ESKD) disproportionately affects certain subgroups of the population in the United States, with preventable differences evident across risk factors, access to care, treatment outcomes, and overall quality of life. Health disparities amongst individuals with ESKD often stem from historical and systemic structures linked to socioeconomic disadvantage, structural vulnerability, and limited access to care (1–3). According to the federal agency, Centers for Medicare and Medicaid Services (CMS), home dialysis and kidney transplantation represent the most effective treatments for many patients with ESKD, yet individuals from at-risk communities who are more likely to have ESKD, are least likely to have access to these evidence-based treatment modalities (4).

In response to inequities in ESKD care, the Center for Medicare and Medicaid Innovation (CMMI), an organization within CMS charged with improving and innovating healthcare payment models, introduced several payment models that emphasize the importance of addressing both clinical and non-clinical factors in kidney care, which include the ESRD Treatment Choices (ETC) Model and the Kidney Care Choices (KCC) Model, amongst others. These models aim to improve access to care and patient outcomes by incentivizing providers and medical centers who adopt comprehensive kidney health management strategies through increasing Medicare payments for kidney disease prevention, promoting the use of home dialysis, and enhancing access to kidney transplantation (4, 5).

Despite these incentives, safety-net systems and Federally Qualified Health Centers (FQHCs), which serve patients who are disproportionately affected by kidney disease, face significant challenges in achieving performance-based metrics due in part to a higher baseline level of patient need and lower overall resource availability (6, 7). Addressing the underlying gaps and non-medical barriers to kidney care is required for these community-based health systems to effectively implement the new CMS payment models. For instance, patients may initiate in-center dialysis without receiving sufficient education on potential alternative treatments, such as home dialysis or preemptive transplantation (8). Additionally, individuals with limited cognitive or physical abilities often lack directed assessments and adequate support, potentially hindering their ability to safely access home dialysis or achieve eligibility for kidney transplantation (9).

Vulnerable patients experiencing ESKD commonly report a range of barriers to high quality care and related needs including, but not limited to, financial insecurity, employment barriers, transportation barriers, food insecurity, mobility limitations, housing insecurity, lack of kidney care knowledge, and cognitive decline (10–13). For this study, we focus on these *non-medical* barriers and have categorized them into three broad domains: social needs, which encompasses social determinants of health (SDOH) factors such as unreliable transportation, financial instability, and access to needs such as food or clothing; cognitive function, including memory difficulties and difficulties with decision-making and planning (often referred to as executive functioning); and functional mobility status, such as functional mobility limitations and difficulties with activities of daily living (ADLs), including dressing, bathing, and basic ambulation. Addressing the intersection of these domains may be key for understanding challenges that individuals with ESKD face and is essential for improving health outcomes and maintaining patient independence.

These challenges are further compounded by differences in the scope of insurance coverage, which represents another significant non-medical driver of health. In the US there are two types of public insurance offered to US residents: for people with limited income or resources, Medicaid is offered by the federal government and managed by each state's government; Medicare is offered to people over the age of 65, or if under the age of 65 with long-term disabilities, ESKD, or Amyotrophic Lateral Sclerosis. Dual-eligible individuals are people who meet requirements for both Medicare and Medicaid, based on socioeconomic status and disability status or age; Medicaid and Medicare are both managed by CMS. Whereas private insurance is typically offered through full-time employment or privately paid for by individuals. Dual-eligible individuals often experience a greater burden of comorbidities compared to those with private insurance (14–16). Individuals with ESKD who are Dual-eligible are disproportionately reliant on in-center dialysis and face reduced access to home-based treatments or kidney transplantation (17). These inconsistencies in care contribute to and likely exacerbate disparities in kidney health outcomes, underscoring the need for tailored policies and interventions to address the unique challenges faced by this population.

To better understand these interconnected barriers to care, the current exploratory, observational study builds upon our previous qualitative research (10). As part of ongoing quality improvement efforts at a large, multi-site dialysis provider in the United States (DaVita), a quantitative survey was developed based on themes identified in our previous publication (10), which explored various needs of individuals undergoing dialysis. The previous qualitative survey focused on social determinants of health (SDOH) needs, ADLs, mobility, cognition, transportation, and social isolation.

In the current large-scale survey of individuals, over the age of 18, currently receiving dialysis, we investigated additional barriers, including cognition, activities of daily living (ADLs), mobility, and social needs. Our objective for this study was to expand upon our previous qualitative study (10) in order to make recommendations for identified areas that present an opportunity for intervention to bridge the gap between the ideal standards of ESKD care and the realities faced by patients in their daily lives.

## Materials and methods

### Survey distribution

The survey was developed from multiple validated health metrics measures and reviewed by the organization's institutional review board (IRB) and programmed by the organization's research staff into HIPAA compliant, Qualtrics survey software (Supplementary Materials). The survey was distributed via electronic mail by the organization to 12,891 of their patients currently receiving care at several of the organization's dialysis centers across the US. After sending the emails, 2,603 emails were rejected, and four email addresses were duplicates; these were removed. The survey was successfully delivered to 10,284 individuals, representing a broad demographic spectrum. Participants were required to indicate consent to participation before being able to access the survey. Patients under the age of 18 were excluded. The survey was open for one month from April 1 to May 1, 2024, and was available in English and Spanish. Blinded and anonymized raw data were provided to the authors by the organization.

### Statistical analyses

For this exploratory observational sequential design study, descriptive, Spearman's correlation, and logistic regression analyses were conducted in R and Jamovi (R v4.3.2; v2.5) and analyzed between June and December 2024. Pearson's Chi-Square test with continuity correction, logistic regressions were additionally performed October through November 2025 utilizing R (v. 4.5.1).

To examine the relationship between insurance type and number of unmet needs, a Pearson's Chi-Square Test was performed, and Spearman's correlations were used to select the predictors for multinomial logistic regression analyses. Multinomial logistic regression was then used to analyze the relationship between the insurance type (dependent variable) and selected predictors.

For the domains of social needs, functional mobility, and cognitive function, logistic regressions using binomial link function were applied to explore the relationship between these dependent variables and predictors. Social needs had eight items with yes/no responses, assessing social determinants of health factors. These questions were added up and recoded as 0 = no for all eight questions, 1 = yes for any of the eight items. For cognitive function, there were four items assessing memory, executive function, and understanding; items were added up and recoded as 0 = no on all four items, and 1 = yes on any of the four items. Similarly, for functional mobility, there were 17 items assessing ADLs and need for mobility aids. Functional mobility items were added up and recoded as 0 = no for all 17 items, 1 = yes for any of the 17 items.

## Results

### Demographics

A total of 962 participants (9.4% of surveys distributed) completed the survey, with a mean age of 65.2 years (SD = 14.4), median age 66, ranging from 21 to 95 years (Table 1). The racial and ethnic composition included 49.3% identifying as White (*n* = 474), 16.2% as Latinx/Hispanic (*n* = 156), 12.8% as Asian American (*n* = 123), 9.7% as Black/African American (*n* = 93), 2.5% as Native American/Alaska Native (*n* = 24), 2% as Native Hawaiian/Pacific Islander (*n* = 19), and 3.1% as Other (*n* = 30). Education attainment was reported as 38.5% (*n* = 370) completing some college, 20.9% (*n* = 201) completing a 4-year degree, 14.9% (*n* = 143) completing a graduate degree, 17.9% (*n* = 172) completing high school, and 5.7% (*n* = 55) with less than a high school education (Table 1). For insurance utilization (Table 2), the majority of respondents reported having Medicare only (*n* = 452, 48.2%), while 36.6% (*n* = 343) had private insurance only or private insurance and Medicare. Dual-eligible participants made up 9.6% (*n* = 90), 5.2% (*n* = 49) had Medicaid only, and 0.4% (*n* = 4) reported being uninsured.

**Table 1:** Demographics.

Participants	*N* = 962
Age (year)	
Range	21–95
Mean (SD)	65.2 (14.4)
Median	66
Race/Ethnicity	*n* (%)
Asian American	123 (12.8%)
Black/African American	93 (9.7%)
Latinx/Hispanic	156 (16.2%)
Native Hawaiian/Pacific Islander	19 (2%)
Native American/Alaska Native	24 (2.5%)
White	474 (49.3%)
Other/multiracial	30 (3.1%)
Did not answer	43 (4.5%)
Education	*n* (%)
Less than high school	55 (5.7%)
High school diploma	172 (17.9%)
Some college	370 (38.5%)
4-year degree	201 (20.9%)
Graduate degree	143 (14.9%)
Did not answer	21 (2.2%)

**Table 2:** Frequencies of insurance type.

Insurance	Counts (*n* = 938)	% of total	Cumulative %
Uninsured	4	0.4%	0.4%
Dual (Medi/Medi)	90	9.6%	10.0%
Medicaid	49	5.2%	15.2%
Medicare	452	48.2%	63.4%
Private Insurance	343	36.6%	100.0%

### Social needs

**Figure 1:**
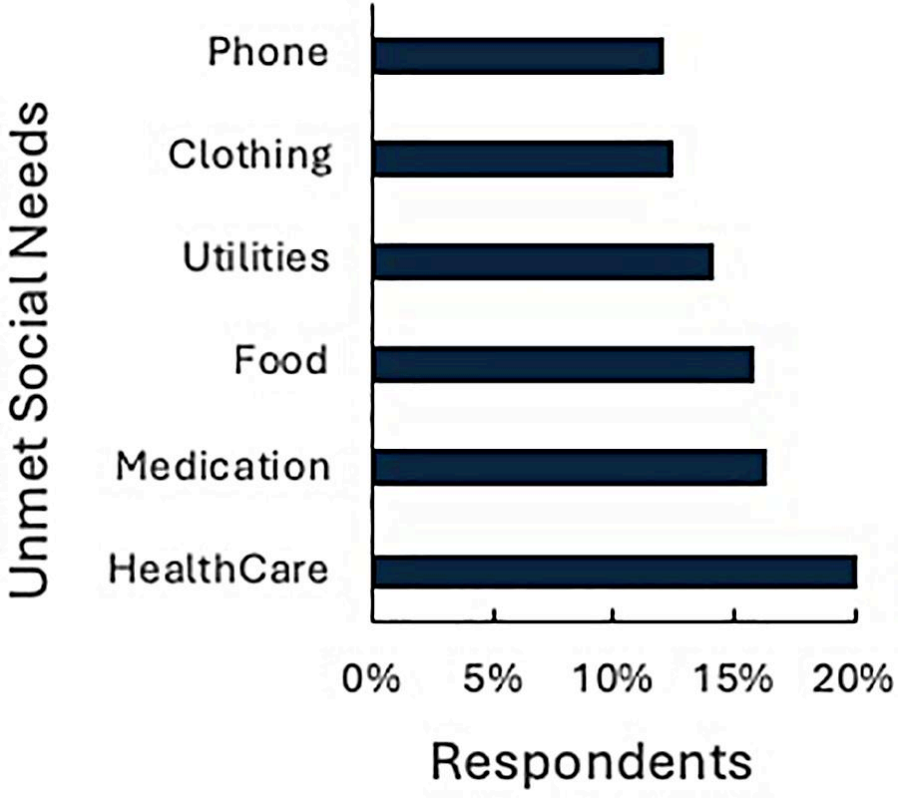
Unmet social needs. Percent of surveyed patients on dialysis who indicated experiencing difficulty accessing basic social needs.

The survey revealed substantial unmet social needs amongst patients receiving dialysis, with over one-third of Dual-eligible participants experiencing three or more unmet social needs (Table 3). A total of 20.4% (*n* = 196) of respondents reported not being able to access healthcare and 16.3% (*n* = 157) indicated inability in obtaining prescribed medications. Food insecurity was reported by 15.8% (*n* = 152) of participants and 14.1% (*n* = 136) experienced inability to pay for utilities, 12.5% (*n* = 120) reported a lack of adequate clothing, and 12.1% (*n* = 117) did not have consistent telephone access (Figure 1). Additionally, 15.7% (*n* = 151) reported having social interactions (e.g., seeing friends/family, talking to friends/family on the phone, attending church) less than once a week and 22.6% (*n* = 218) reported having social interactions one or two times a week.

**Table 3:** Prevalence of multiple unmet social needs across insurance type.

Insurance type	Number of unmet social needs *N* (% of total for insurance type)			
	0	1–2	3–4	5+
Medicaid	29 (60%)	8 (17%)	10 (19%)	2 (4%)
Medicare	220 (47%)	119 (27%)	90 (21%)	23 (5%)
Dual Eligible	32 (36%)	25 (28%)	22 (24%)	11 (12%)
Private Insurance	186 (67%)	76 (21%)	36 (10%)	6 (2%)
None/Uninsured	1 (25%)	1 (25%)	1 (25%)	1 (25%)

*χ*^2^ test of association *p* < 0.05.

In the logistic regression (Table 4), it was revealed that participants with unmet healthcare needs were more likely to have public insurance (OR: 2.687–4.076, *p* < 0.05) than private insurance. Participants who had fewer social interactions in a week were more likely to have Medicare (OR: 1.214, *p* < 0.05) or be Dual-eligible (OR: 1.357, *p* < 0.05) insurance recipients. Furthermore, participants who indicated concern about personal safety where they lived were more likely to also have a higher number of unmet social needs (Table 5; OR: 3.667, *p* < 0.05).

**Table 4:** Odds ratios for insurance type by predictor.

Predictors	Insurance type					
	Medicare OR (95% CI)	*p*-value	Medicaid OR (95% CI)	*p*-value	Dual OR (95% CI)	*p*-value
Age	1.020 (1.005, 1.036)	0.009**	0.956 (0.934, 0.978)	0.000***	0.980 (0.957, 1.005)	0.113
Education	0.866 (0.725, 1.034)	0.112	0.647 (0.474, 0.884)	0.006**	0.588 (0.426, 0.810)	0.001***
Employment	0.685 (0.506, 0.926)	0.014*	0.699 (0.447, 1.093)	0.116	0.756 (0.446, 1.281)	0.299
Childcare needs	0.271 (0.107, 0.691)	0.006**	0.164 (0.040, 0.674)	0.012*	0.000 (0.000, 0.000)	0.000***
Healthcare needs	2.686 (1.431 5.043)	0.002**	4.946 (2.134, 11.461)	0.000***	4.076 (1.727, 9.618)	0.001***
Social interaction/isolation	1.214 (1.432, 1.439)	0.025*	0.990 (0.740, 1.323)	0.944	1.357 (1.016, 1.812)	0.039*
ADL—feeding self	0.675 (0.236,1.931)	0.464	0.476 (0.081, 2.799)	0.411	0.581 (0.121, 2.797)	0.498
ADL—bathing self	0.533 (0.240, 1.184)	0.122	0.207 (0.039, 1.103)	0.065	0.622 (0.193, 2.000)	0.425
ADL—dressing upper body	1.341 (0.375, 4.791)	0.652	8.222 (1.019, 66.366)	0.048*	4.096 (0.659, 25.469)	0.130
ADL—dressing lower body	1.481 (0.457, 4.793)	0.512	1.513 (0.237, 9.656)	0.662	0.835 (0.149, 4.689)	0.838
ADL—toileting	1.422 (0.335, 6.035)	0.633	0.654 (0.076, 5.611)	0.698	0.624 (0.077, 5.033)	0.658
Use wheelchair	1.725 (0.844, 3.528)	0.135	1.801 (0.480, 6.749)	0.383	1.554 (0.546, 4.423)	0.409
Use hospital chair	1.848 (0.310, 10.997)	0.500	4.806 (0.390, 59.205)	0.220	2.345 (0.225, 24.476)	0.476
Use cane	1.235 (0.737, 2.069)	0.424	1.320 (0.523, 3.333)	0.557	1.938 (0.869, 4.321)	0.106
Use hospital bed	1.172 (0.289, 4.760)	0.824	0.971 (0.136, 6.949)	0.977	0.491 (0.061, 3.926)	0.502
Use walker	0.809 (0.442, 1.482)	0.493	0.332 (0.104, 1.058)	0.062	1.395 (0.541, 3.600)	0.491
Use toilet chair	0.783 (0.345, 1.780)	0.559	1.490 (0.334, 6.651)	0.601	1.823 (0.574, 5.783)	0.308
Use shower chair	1.474 (0.829, 2.619)	0.187	1.685 (0.620, 4.577)	0.306	1.510 (0.609, 3.746)	0.374
Use car transfer device	7.170 (0.765, 67.229)	0.085	4.357 (0.250, 75.931)	0.313	5.520 (0.340, 89.556)	0.229
Difficulty with chores	1.141 (0.883, 1.473)	0.313	0.904 (0.578, 1.414)	0.658	0.968 (0.622, 1.507)	0.887
Difficulty walking up stairs	1.074 (0.848, 1.361)	0.554	1.536 (1.030, 2.291)	0.035*	1.286 (0.872, 1.896)	0.204
Difficulty running errands	0.788 (0.609, 1.020)	0.071	0.989 (0.626, 1.560)	0.961	0.983 (0.645, 1.497)	0.936
Difficulty walking 15 min	0.987 (0.788, 1.238)	0.913	0.703 (0.462, 1.069)	0.099	0.810 (0.551, 1.191)	0.285
Difficulty remembering things	0.960 (0.699, 1.319)	0.802	0.706 (0.420, 1.187)	0.189	1.233 (0.754, 2.018)	0.403
Difficulty planning/making decisions	1.016 (0.713, 1.448)	0.928	1.976 (1.164, 3.353)	0.012*	1.007 (0.594, 1.706)	0.979
Difficulty understanding kidney disease	1.082 (0.888, 1.318)	0.435	0.868 (0.612, 1.230)	0.426	0.844 (0.575, 1.239)	0.386
Difficulty making decisions about kidney treatment	0.909 (0.701, 1.180)	0.473	0.970 (0.624, 1.507)	0.892	1.018 (0.653, 1.587)	0.937

* *p* < 0.05, **p < 0.01, ***p < 0.001.

**Table 5:** Odds ratio for social needs by predictor.

Predictor	OR (95% CI)	*P* value
Age	0.977 (0.961, 0.992)	0.003**
Education	0.846 (0.687, 1.035)	0.105
Employment	0.954 (0.677, 1.331)	0.783
Insurance type	1.295 (1.027, 1.633)	0.028*
Transportation	0.470 (0.197, 1.132)	0.088
Social interaction/isolation	1.197 (0.985, 1.455)	0.069
Personal safety	3.667 (1.215, 11.588)	0.022*
ADL—feeding	1.900 (0.589, 6.080)	0.277
ADL—bathing	0.666 (0.259, 1.600)	0.377
ADL—dressing upper body	1.468 (0.399, 5.644)	0.567
ADL—dressing lower body	0.701 (0.193, 2.329)	0.573
ADL—toileting	0.594 (0.133, 2.410)	0.477
Use wheelchair	0.705 (0.313, 1.539)	0.387
Use hospital chair	0.427 (0.054, 2.242)	0.354
Use cane	1.321 (0.739, 2.346)	0.344
Use hospital bed	1.474 (0.445, 4.668)	0.511
Use walker	0.639 (0.315, 1.273)	0.207
Use toilet chair	2.128 (0.919, 4.889)	0.075
Use shower chair	1.082 (0.574, 2.015)	0.805
Use car transfer device	0.762 (0.108, 3.935)	0.761
Difficulty with chores	1.259 (0.943, 1.681)	0.117
Difficulty walking up stairs	1.016 (0.781, 1.323)	0.906
Difficulty running errands	0.992 (0.744, 1.323)	0.954
Difficulty walking 15 min	0.863 (0.661, 1.119)	0.270
Difficulty remembering things	1.292 (0.924, 1.807)	0.134
Difficulty planning/making decisions	0.978 (0.678, 1.394)	0.905
Difficulty understanding kidney disease	1.186 (0.956, 1.464)	0.115
Difficulty making decisions about kidney treatment	1.029 (0.780, 1.353)	0.840

* *p* < 0.05, **p < 0.01.

### Functional mobility status

**Figure 2:**
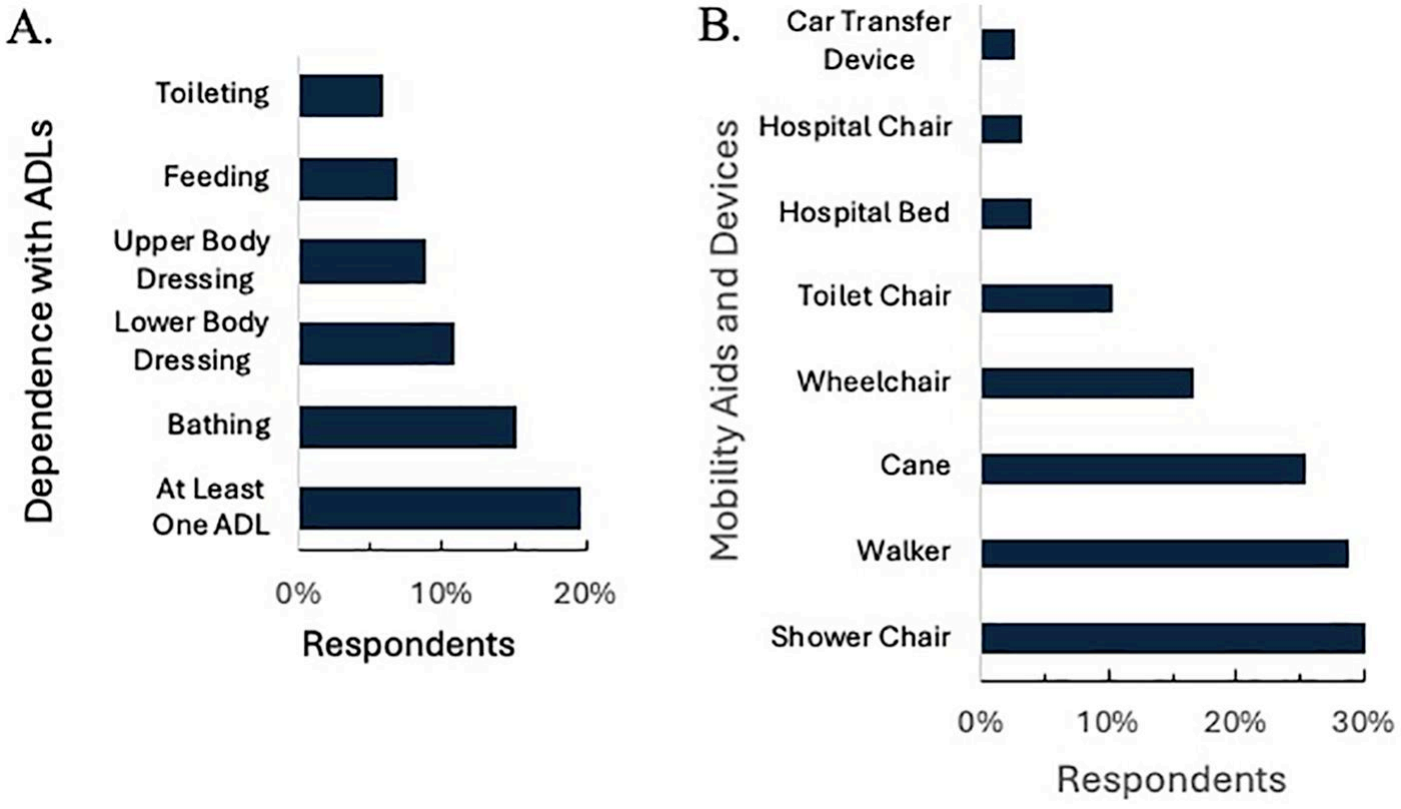
Functional limitations. Participants were surveyed about their experiences across three areas of Activities of daily life (ADL): **(A)** whether they depended on other for ADLs, **(B)** used specific mobility aids and devices.

Nearly one-fifth (19.6%; *n* = 189) of participants reported needing assistance with at least one ADL, including feeding, bathing, dressing, and toileting (Figure 2A). Over two-fifths (41.6%; *n* = 401) of participants utilized ambulatory aids such as wheelchairs, canes, or walkers, while 32.9% (*n* = 317) used aids for ADLs, including car transfer devices, toilet chairs, or shower chairs (Figure 2B).

**Figure 3:**
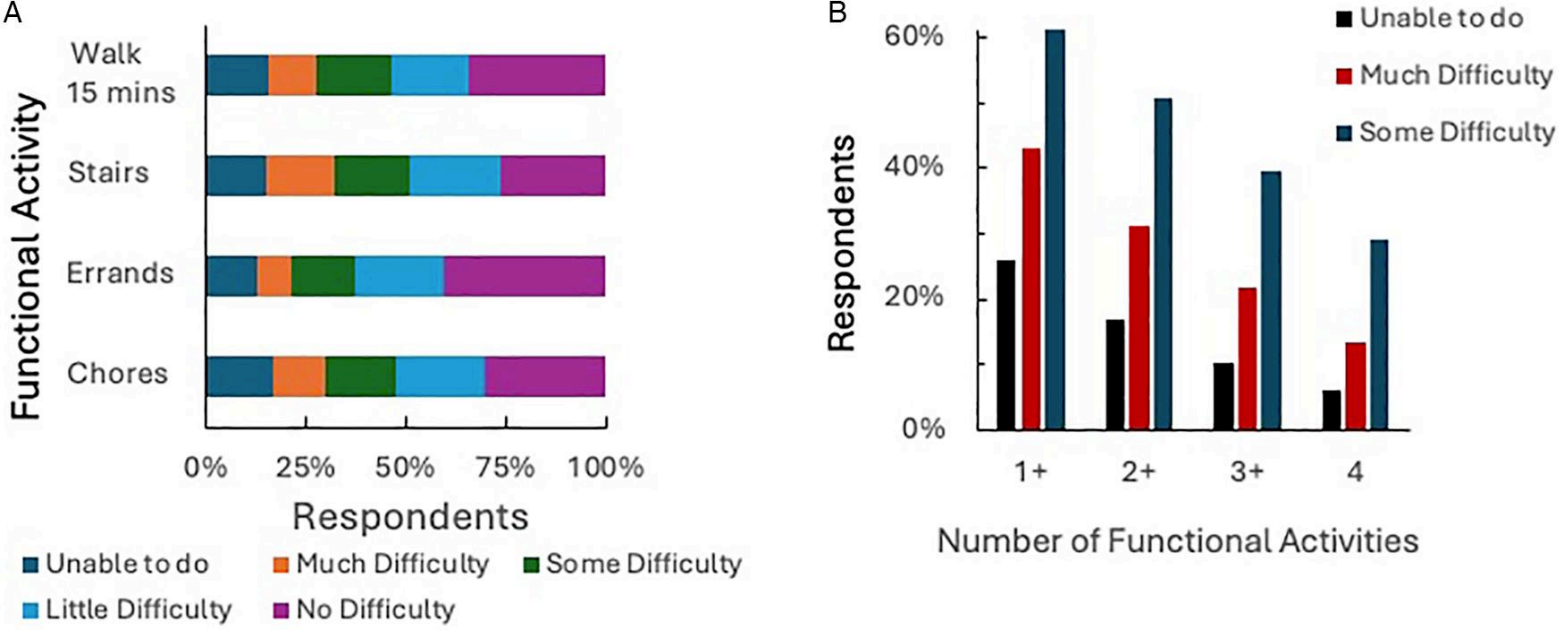
Functional activities **(A)** and amount of difficulty required to complete activities that require mobility, **(B)** percentage of participants who experienced difficulty with one or more functional activities.

To assess mobility limitations, we asked participants whether they experienced difficulties walking up one flight of stairs, performing household chores, walking for 15 min, or running errands. More than half (51.0%; *n* = 485) of respondents experienced at least some difficulty performing two or more of these activities, with 31.1% (*n* = 299) reporting much difficulty or inability to complete the activities at all (Figures 3a,b). The logistic regression (Table 4) revealed that participants with difficulty dressing the upper body (OR: 8.222, *p* = 0.048) or more difficulty walking up a flight of stairs (OR: 1.536, *p* = 0.035) were significantly more likely to have Medicaid than private insurance. Participants who reported difficulty with memory (Table 6), were also more likely to have functional mobility difficulties (OR: 2.786, *p* = 0.000), while participants who were employed were significantly less likely to experience functional mobility difficulties (OR: 0.640, *p* = 0.010).

**Table 6:** Odds ratio for functional mobility by predictor.

Predictor	OR (95% CI)	*P* value
Age	1.028 (1.010, 1.046)	0.002**
Education	0.883 (0.700, 1.111)	0.289
Employment	0.640 (0.454, 0.903)	0.010*
Insurance Type	1.657 (1.203, 2.332)	0.003**
Transportation	0.368 (0.055, 1.414)	0.205
Social Interaction/Isolation	1.067 (0.854, 1.340)	0.569
Personal Safety	3.78E + 06 (1.07E + 26, 8.54E + 149)	0.9855
Difficulty Remembering Things	2.786 (1.633, 4.990)	0.000***
Difficulty Planning/Making Decisions	11.371 (0.742, 2.895)	0.359
Difficulty Understanding Kidney Disease	0.935 (0.735, 1.205)	0.589
Difficulty Making Decisions About Kidney Treatment	1.202 (0.838, 1.767)	0.331
Unmet Food Needs	0.585 (0.147, 2.737)	0.466
Unmet Clothing Needs	1.090 (0.196, 6.821)	0.923
Unmet Utilities Needs	0.909 (0.250, 3.625)	0.888
Unmet Childcare Needs	2.236 (0.449, 11.461)	0.323
Unmet Medication Needs	1.003 (0.307, 3.544)	0.996
Unmet Healthcare Needs	1.570 (0.544, 5.258)	0.430
Unmet Phone Needs	0.685 (0.113, 4.140)	0.677
Other Unmet Needs	0.906 (0.268, 3.464)	0.879

* *p* < 0.05, **p < 0.01, ***p < 0.001.

### Cognitive function

**Figure 4:**
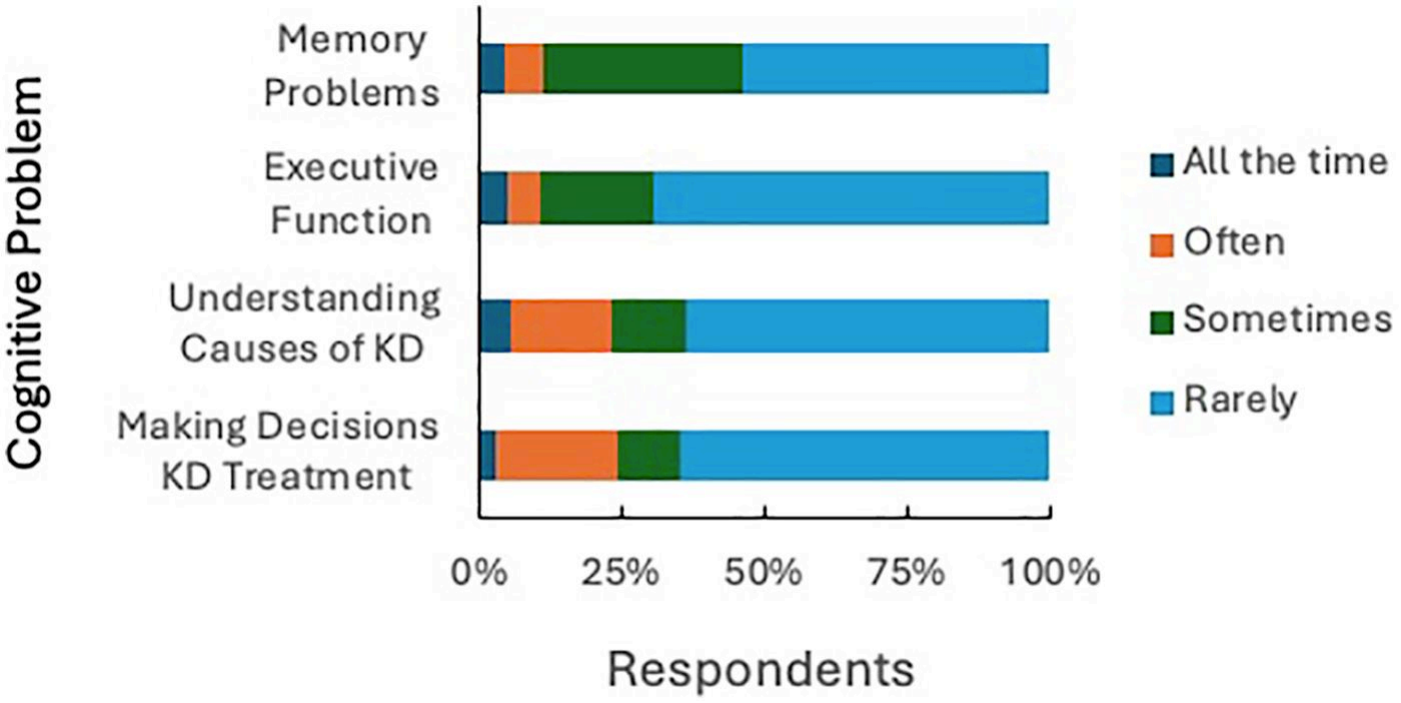
Cognitive function. Perceived difficulty across cognitive tasks.

Cognitive challenges were prevalent, with 45.1% (*n* = 435) of respondents reporting experiencing memory problems (i.e., “unable to remember things that happened or what people told you”) at least sometimes, 34.8% (*n* = 336) expressed sometimes, often, or always having difficulty understanding the causes of their kidney disease, 34.5% (*n* = 332) reported sometimes, often, or always having difficulty making decisions about kidney treatment, and 29.7% (*n* = 286) indicating difficulty with executive functioning skills (i.e., planning ahead, making appointments, making decisions) (Figure 4).

In a logistic regression (Table 4), individuals who reported difficulty with planning ahead, making appointments, or making decisions were significantly more likely to have Medicaid than private insurance (OR: 1.976, *p* = 0.012). Participants who reported difficulty walking up a flight of stairs (OR: 1.296, *p* = 0.04) and unmet food needs (OR: 4.000, *p* = 0.047) had significantly more cognitive function difficulties, while participants reporting mobility difficulties requiring a toilet chair (OR: 0.373, *p* = 0.024) or childcare needs (OR: 0.234, *p* = 0.040) experienced significantly fewer cognitive function difficulties (Table 7).

**Table 7:** Odds ratio for cognitive function by predictor.

Predictor	OR (95% CI)	*P* value
Age	0.995 (0.980, 1.010)	0.521
Education	0.9400 (0.777, 1.137)	0.523
Employment	0.816 (0.597, 1.114)	0.199
Insurance type	1.265 (0.996, 1.618)	0.057
Transportation	1.2613 (0.417, 3.431)	0.661
Social interaction/isolation	1.174 (0.977, 1.413)	0.088
Personal safety	2.127 (0.511, 14.713)	0.356
ADL—feeding	0.777 (0.184, 4.143)	0.744
ADL—bathing	1.379 (0.561, 3.610)	0.496
ADL—dressing upper body	6.989 (1.020, 68.089)	0.062
ADL—dressing lower body	0.624 (0.135, 3.266)	0.556
ADL—toileting	3.488 (0.416, 78.594)	0.311
Use wheelchair	0.959 (0.437, 2.168)	0.919
Use hospital chair	0.365 (0.058, 3.114)	0.301
Use cane	1.367 (0.768, 2.466)	0.292
Use hospital bed	7.379 (1.048, 163.615)	0.095
Use walker	0.742 (0.377, 1.459)	0.386
Use toilet chair	0.373 (1.57, 0.883)	0.024*
Use shower chair	1.209 (0.660, 2.237)	0.541
Use car transfer device	1.573 (0.209, 33.028)	0.700
Difficulty with chores	0.922 (0.696, 1.220)	0.567
Difficulty walking up stairs	1.296 (1.013, 1.664)	0.040*
Difficulty running errands	1.081 (0.816, 1.435)	0.589
Difficulty walking 15 minutes	1.194 (0.939, 1.523)	0.150
Unmet food needs	3.996 (1.106, 17.584)	0.047*
Unmet clothing needs	0.2669 (0.048, 1.303)	0.112
Unmet utilities needs	2.924 (0.900, 11.221)	0.090
Unmet childcare needs	0.234 (0.055, 0.899)	0.040*
Unmet medication needs	1.024 (0.408, 2.596)	0.960
Unmet healthcare needs	0.810 (0.370, 1.825)	0.602
Unmet phone needs	1.355 (0.284, 6.991)	0.706
Other unmet needs	1.269 (0.466, 3.690)	0.649

* *p* < 0.05.

### Stratification by insurance status

Across multiple public and private insurance types, the survey results showed pronounced disparities in unmet needs, with Medicaid and Dual-eligible participants reporting the highest burden of unmet needs compared to those with Medicare and private insurance (Table 3). Medicare was the primary insurance provider for 48.2% of insurance respondents (*n* = 452 out of 938) (Table 2). Among all Medicare-only recipients, 17.7% reported two or more unmet needs, including 9.7% with five or more unmet needs. In stratifying Dual-eligible participants, 25.0% experienced two or more unmet needs and 12.0% with five or more unmet needs. In contrast, respondents with private insurance reported markedly fewer unmet needs, with 61.2% experiencing no unmet needs and only 17.5% reporting two or more (Table 3).

Participants across all public insurance types were more likely to experience unmet healthcare needs than with private insurance (Table 4; OR: 2.686–4.946; *p* < 0.05). Participants of higher age were more likely to have Medicare insurance (OR: 1.020, *p* = 0.009), while younger participants were more likely to have Medicaid (OR: 0.956, *p* = 0.000). Participants with public insurance were more likely to have a higher number of unmet social needs than people with private insurance (Table 5; OR: 1.295, *p* = 0.028) and participants who reported feeling unsafe where they live (OR: 3.667, *p* = 0.022) were also more likely to have a higher number of unmet social needs. Participants who reported being unemployed and seeking work were significantly less likely to have Medicare (Table 4; OR: 0.685, *p* < 0.05) and participants with lower educational attainment were more likely to be on Medicaid (OR: 0.647, *p* = 0.006) or be Dual-eligible (OR: 0.588, *p* = 0.001). Of note, participants who reported experiencing social isolation were more likely to have Medicare (OR:1.214, *p* = 0.025) or be Dual-eligible (OR: 1.357, *p* = 0.039), similar to findings from our previous study focused on Dual-eligible patients (10).

### Overlapping challenges

**Figure 5:**
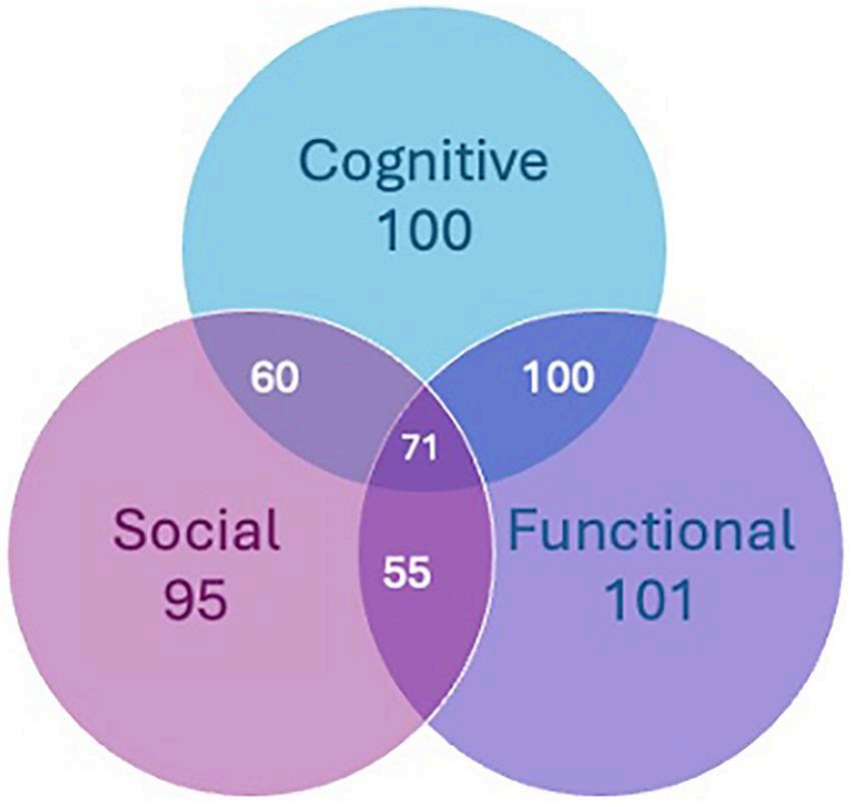
Intersection of cognitive decline, social needs, and functional limitations. Number of respondents indicating cognitive problems often or all of the time (Cognitive, blue); facing at least two ADL limitations or 1 functional mobility limitation (Functional, purple); or experiencing at least one unmet social need (Social, pink). 15.9% (*n* = 100) experienced both cognitive and functional challenges, 5.7% (*n* = 55) indicated both functional limitations and unmet social needs, 6.2% (*n* = 60) experienced both cognitive decline and unmet social need, and 7.4% (*n* = 71) faced challenges across all three domains.

Out of the total sample, 60.4% (*n* = 582) participants indicated experiencing one or more difficulties with cognition, unmet social needs, and/or functional limitations. Of this subset, participants 56.9% (*n* = 331) reported experiencing cognitive challenges often, or always/all the time, 22.5% (*n* = 131) experienced cognitive limitations and unmet social needs, 29.4% (*n* = 171) experienced both cognitive challenges and functional limitations, 12.2% (*n* = 71) experienced cognitive challenges, unmet socials needs, and functional limitations (Figure 5). Memory challenges also predicted more mobility difficulties and vice versa (OR: 2.786, *p* = 0.000; Table 6 and OR: 1.296, *p* = 0.040; Table 7), while employment negatively predicted functional difficulties (OR: 0.640, *p* = 0.010).

## Discussion

Our findings revealed a notable convergence of non-medical barriers categorized as unmet social needs, functional mobility limitations, and cognitive function difficulties among ESKD patients undergoing dialysis in the US; these overlapping factors compound complexity to patient care delivery. The survey also highlighted substantial unmet social determinants of health (SDOH) needs associated with insurance type. To our knowledge no other studies have examined the relationship between people undergoing dialysis and cognition, social needs, functional mobility, alongside insurance type. Although other studies have examined some of these conditions in conjunction with dialysis, none to our knowledge have examined all of these factors together.

In the present study, the majority of participants relied on public insurance (61.4%), experienced unemployment (72.7%), and/or identified as Asian American, Black/African American, Latinx/Hispanic, Native Hawaiian/Pacific Islander, Native American/Alaska Native, or Other (50.7%). These findings align with prior research demonstrating that underserved patients with unmet social needs significantly influence outcomes for patients receiving dialysis and decrease likelihood of receiving a transplant (18–23). Based upon our data, it appears that patients with private insurance had the fewest unmet needs (67%), while the majority of participants who were Dual-eligible had one or more unmet needs.

### Cognitive challenges

Despite being well documented, cognitive challenges amongst patients receiving dialysis are frequently underdiagnosed and overlooked despite their substantial impact on patient outcomes (24–26). Cognitive dysfunction is common in patients with ESKD, with the rate of cognitive decline positively correlated with worsening renal function (9, 25–27). Furthermore, cognitive decline related to ESKD significantly increases the risk of developing mild cognitive impairment (MCI) and major neurocognitive disorder (28–30). While estimates of cognitive impairment prevalence amongst patients with chronic kidney disease (CKD) and ESKD range from 20% to 70%, it is believed to be underdiagnosed in over 80% of cases (30, 31).

In line with previous studies, indicating that ESKD affects cognitive functions including memory and executive functions (32, 33), in our sample of individuals undergoing dialysis treatment, 45.1% of respondents reported difficulties with memory, while approximately one-third reported difficulty understanding reasons for kidney disease (34.8%), difficulty understanding treatment (34.4%), and difficulty planning ahead, making appointments, or making decisions, referred to as executive functioning skills (29.7%).

Cognitive dysfunction often compounds non-medical treatment challenges, such as health literacy challenges, understanding reasons for treatment, or being able to plan for treatments accordingly, and represents an additional substantial barrier to effective treatment decision-making and clinical service delivery. Furthermore, cognitive dysfunction may preclude kidney transplant listing (9).

### Functional independence

In our study, more than half of respondents reported significant mobility issues, with many requiring assistance for basic tasks. Over one-third (*n* = 326; 33.6%) of the participants experienced both ADL and mobility impairments, highlighting the strong connection between physical ability and functional limitations. Added to the difficulties related to cognitive challenges stemming from ESKD, functional limitations further complicate care and may significantly impact treatment outcomes.

Functional independence, or the ability to perform ADLs across different ages and patient populations, is strongly associated with greater health-related quality of life (34–36). A loss of functional independence has been linked to poorer outcomes in older patients and is associated with increased all-cause mortality risk (37, 38). The co-occurrence of cognitive decline and physical limitations amongst ESKD patients increases the complexity of their care needs and may contribute to a preference for clinic-based dialysis over in-home options (8). To address these challenges effectively, providers must assess for physical/functional mobility and cognitive abilities, and treatment-related complications to tailor treatment recommendations appropriately (39).

For example, one intervention, the EXCITE (Extremity Constraint Induced Therapy Evaluation) trial, tested an at-home exercise program that consisted of two 10-minute walks each non-dialysis day for 6-months and showed a 29% reduction in combined risk of death and hospitalization after 36 months (40, 41). Patients who closely adhered to the program experienced the most risk reduction. In our study, we found that 74% of participants were able to complete a 15 min walk with some- to no-difficulty, indicating that this intervention would likely be appropriate for most patients receiving dialysis. Prescribed walking with regular follow-ups could be a highly successful strategy for increasing functional independence and decreasing overall risk.

While certain pharmacologic interventions may slow cognitive decline, non-pharmacologic interventions such as physical exercise have been shown to improve not only cognition but also mobility (42–45), even for people who have CKD (46, 47). Increased physical activity would not only improve functional independence, but may also reduce the risk of comorbidities, such as cardiovascular disease, frailty, or hospitalization, while improving overall quality of life for patients with CKD/ESKD. To promote functional independence, referrals to physical or occupational therapists specializing in chronic medical conditions should be routinely implemented (48, 49).

### Social isolation

In the current study, more than one in seven participants (15.7%; *n* = 151) reported having limited social interactions (less than once a week) and were more likely to be experienced by Medicare and Dual-eligible participants (OR: 1.214–1.357 respectively, *p* < 0.05) than those with private insurance. In our previous study surveying Dual-eligible participants, interviewees reported experiencing social isolation as a result of their ESKD and associated disabilities, and that they utilized in-center dialysis as a means of social contact with other people (10).

Social isolation has been shown to be a risk factor for increased depressive symptoms, worse overall outcomes, as well as being a risk factor for neurocognitive disorders and cognitive decline (50–53). In a large cohort study examining social support, it was found that more than one in five people with predialysis CKD experienced low social support (54). Implementing interventions that would increase social interactions for people with ESKD, such as prescribed group exercise, would improve mobility while decreasing social isolation, and potentially also mitigating cognitive dysfunction (46, 47). Moreover, similar to neuroprotective effects of exercise, social interactions have also been associated with increased cognitive reserve and slowing of cognitive decline (44, 55).

### Health literacy

Although our study did not specifically assess for health literacy, we did include questions asking participants whether they had difficulty understanding the causes of kidney disease and if they had difficulty making decisions about their kidney treatment. Over one-third of participants (34.8% and 34.5%, respectively) indicated at least sometimes having difficulty understanding the causes of kidney disease or making decisions about kidney treatment, which could be in part due to low health literacy.

Lack of health literacy about CKD/ESKD and treatment in individuals undergoing dialysis has been a commonly identified challenge associated with poorer health outcomes (56) and less likelihood of transplantation (57, 58). Utilization of existing patient- or person-centered care strategies, such as collaborative care planning, motivational interviewing, “teachback” method, and other techniques may improve dialysis patients’ understanding, engagement with treatment options or treatment planning, and overall health literacy (59). Furthermore, utilization of interactive training techniques for medical staff, has proven to be effective in training staff to assess for patients with low health literacy, and adjust the way information is presented to patients to ensure understanding (60). Despite recommendations from the National Kidney Foundation (61), there is no standardized method for delivering or assessing for health literacy across healthcare systems.

### Compounding challenges

In the current study, we found multiple, often overlapping, areas of need. The number of cognitive, functional, and social needs challenges was positively correlated with those who were lower resourced and/or low income (e.g., Medicaid and Dual-eligible) in our participant sample. This seems to be similar to previous studies which examined fewer, but similar, factors than the current study (15).

Even though the prevalence of cognitive impairment in CKD/ESKD is well-documented, a reported gap persists between provider and patient perceptions regarding patient abilities, particularly in understanding treatment and self-management of prescription medications or treatment options like home-based self-dialysis (47, 48). These findings are further complicated by the potential association between cognitive and functional mobility challenges, which were elucidated in the current study. This is especially salient because home dialysis requires substantial executive functioning, physical ability, and manual dexterity to perform tasks such as catheter preparation, solution selection, or connecting tubing accurately and safely, as well as keeping ports and catheters sterile. Peritonitis is a significant and relatively common complication of peritoneal dialysis, typically the modality for home dialysis, and is the leading cause of death for people undergoing peritoneal dialysis (64–66). These challenges are exacerbated by increasing cognitive decline.

Basic mobility is essential for performing important tasks, including navigating around the house, preparing meals, bathing, running errands, traveling to medical appointments, and operating home dialysis equipment. Previous research by the authors and others has demonstrated that most patients undergoing dialysis experience restricted mobility (10, 67). Physical capabilities often deteriorate as CKD progresses, further limiting patients’ ability to engage in physical activity (67, 68), which may be a significant risk factor for comorbidities such as cardiovascular disease, type 2 diabetes, hypertension, and mortality amongst patients with ESKD (69–72).

Despite these findings across studies, there is no standardized approach or omnibus method to evaluate these challenges that individuals receiving dialysis treatment may experience. Hall et al. performed a qualitative assessment study examining older adults undergoing dialysis by surveying patients, medical providers, and dialysis staff. The researchers found that participants reported poor mobility assessment, transportation barriers, inappropriate prescribing, poor communication between medical providers, poor communication between providers and patients, and a lack of social and emotional support for people in dialysis treatment (63).

A number of care models for older adults and those with disability or chronic disease have been suggested for adaptation to ESKD, such as the GRACE (Geriatric Resources for Assessment and Care of Elders) care model, involving in-home care and assessment by an advanced practice provider and a social worker (63). One care model that would be of benefit would be an adaptation of the CAPABLE (Community Aging in Place, Advancing Better Living for Elders) care model, which was designed for low income older adults and involves a registered nurse, occupational therapist, and handyman who would provide in-home care, assessment, and home modifications or improvements to help with ADLs and other mobility barriers (73). A small pilot study utilizing the CAPABLE care model adapted to low-income older adults undergoing in-center dialysis called SOCIABLE (Seniors Optimizing Community Integration to Advance Better Living with ESRD), was conducted. The researchers found improvements in ADLs, iADLs, and social support/social network (74), indicating that this type of model may be beneficial for improving the quality of life for older adults on dialysis. Despite these potential solutions that have shown promise for mitigating unmet needs and care gaps, none have been widely adopted.

A prime low-initiation opportunity to address unmet needs, assess for health literacy, and engage patients is during in-center dialysis. Patients undergoing dialysis are typically sitting in one place for several hours each session, several days per week. The dialysis care team could use this this opportune time to assess for patient needs, and to implement additional support services accordingly. However, additional support is needed to optimize dialysis staff's ability to provide tailored patient centered education and follow up. This intentional additive support will ensure that patients better understand kidney health care options and plans and are empowered to make health decisions that provide them with optimal kidney health outcomes.

### Limitations

This study was an exploratory observational sequential design, building upon our previous qualitative study. The lack of hypothesis and measures specifically designed for investigating the relationship between domains may weaken the reported results. The reliance on self-reported data introduces potential biases, including recall bias and social desirability bias, which may impact the accuracy of reported barriers and unmet needs. The use of a convenience sample drawn from patients receiving care at a specific organization may limit the external validity and generalizability of the findings, and the low response rate (9.4%) may also bias the results.

Additionally, the cross-sectional study design restricts the ability to infer causality or examine temporal changes in cognitive, functional, or social determinants of health. Although the survey captured a large, demographically diverse cohort, certain subgroups, such as those who did not have access to email or digital communication, may have been underrepresented, limiting the ability to fully characterize disparities across racial, ethnic, or socioeconomic strata. Furthermore, surveys and raw data were managed by the organization; data provided to the authors were anonymized and limited to exclude any identifying information, including location of dialysis clinics, again potentially skewing results based on geographical characteristics. Finally, although the survey questions were taken from various standardized and validated instruments, put together the survey was not validated for measuring specific domains in individuals with ESKD, such as functional and cognitive limitations or social needs, as a whole, and may impact the comparability of these findings with prior research. Future studies would benefit from validation of standardized assessments measuring the severity of needs across these domains to better stratify the study population and analyses of needs. Future studies employing longitudinal designs and validated measures in broader CKD/ESKD populations are necessary to confirm and extend these findings.

## Conclusion

Given the survey findings, policies for patient-centered kidney care could be adapted to better integrate additional multi-domain assessment and support services into dialysis care, ensuring that patients’ non-medical needs, including health literacy needs, are addressed alongside their medical needs. The significant unmet cognitive, social, and functional needs amongst public insurance recipients in our sample highlight the limitations of current public insurance programs, which often fail to cover the comprehensive care necessary for this vulnerable population.

Future studies should explore these potential areas of deficit to improve access to services, better tailor resource allocation, address non-medical social needs and barriers, as well as inform future medical practices and policies. Furthermore, expanding insurance coverage to include essential social services and increasing the flexibility and comprehensiveness of Medicaid and Medicare benefits, such as individualized assessment to remove barriers to timely transplant listing and improving support for home dialysis, would enhance access to kidney health care, improve kidney health outcomes, and lower overall costs. Ultimately, multi-level, multi-disciplinary interventions are needed to ensure that health systems build services to meet the needs of our most vulnerable patients.

## Data Availability

The datasets presented in this article are not readily available because the data are wholly owned by Davita Inc. and are not available for distribution. Requests to access the datasets should be directed to Victoria Liou-Johnson, vlioujohnson@stanford.edu.
