# Supplementary material for: Cognitive, functional, and social disparities in patients receiving dialysis: a multi-site survey
Source: Front Health Serv. 2026 Jan 20;5:1688966. doi: 10.3389/frhs.2025.1688966 (PMC12864511; doi:10.3389/frhs.2025.1688966)
Supplement: Supplementary file 1 [file Datasheet1.pdf]

[Questions 1-12 from PRAPARE]

1. Which race/races are you? (check all that apply)
    a. Asian
    b. Native Hawaiian
    c. Pacific Islander
    d. Black/African American
    e. White
    f. Latino/Hispanic
    g. American Indian/Alaskan Native
    h. Other (please write):
    i. I choose not to answer this question

2. What language do you use most often? (fill in) ______

3. How many family members, including yourself, do you currently live with? (fill in and multiple choice) _____

    a. I do not live with family members
    b. I do not live with anyone
    c. I choose not to answer this question

4. What is your housing situation today? (choose one)
    a. I have housing
    b. I have housing, but have been unhoused in the past or am worried about losing my housing
    c. I do not have housing (staying with other, in a hotel, in a shelter, living outside on the street, living on a beach, living in a car, or in a park)
    d. I choose not to answer this question

5. What is the highest level of school that you have finished? (choose one)
    a. Less than high school degree
    b. High school diploma or GED
    c. More than high school
    d. Bachelor’s degree
    e. Graduate school
    f. I choose not to answer this question

6. What is your current work situation (choose one)
    a. Unemployed
    b. Part-time or temporary work
    c. Full-time work
    d. Otherwise unemployed but not seeking work (ex: student, retired, disabled, unpaid primary caregiver - please write:______________

e. I choose not to answer this question

7. What is your main insurance? (check all that apply)
   a. none/uninsured
   b. Medicaid
   c. Medicare
   d. Other public insurance
   e. Private insurance

8. In the past year, have you or the people you live with been **unable** to get any of the following when it was **really needed?** (check all that apply)
   a. (Y/N) Food
   b. (Y/N) Clothing
   c. (Y/N) Utilities
   d. (Y/N) Child Care (Y/N)
   e. (Y/N) Medicine or any Health Care (medical, dental, mental health, vision)
   f. (Y/N) Phone
   g. Other (please write): ___________________
   h. I choose not to answer this question

9. Has lack of transportation kept you from medical appointments, meetings, work, or from getting things needed for daily living? (check all that apply)
   a. Yes, it has kept me from medical appointments
   b. Yes, it has kept me from non-medical meetings, appointments, work, or from getting things that I need
   c. No
   d. I choose not to answer this question

10. How often do you see or talk to people that you care about and feel close to? (For example: talking to friends on the phone, visiting friends or family, going to church or club meetings)
   a. Less than once a week
   b. 1 or 2 times a week
   c. 3 to 5 times a week
   d. 5 or more times a week
   e. I choose not to answer this question

11. Do you feel physically and emotionally safe where you currently live?
   a. Yes
   b. No
   c. Unsure
   d. I choose not to answer this question

[Questions 12 -13 adapted from FIM]

12. Do you need assistance with any of these daily activities? (check all that apply)
    a. No, I do not need help
    b. I need help with feeding
    c. I need help with bathing
    d. I need help with dressing upper body
    e. I need help with dressing lower body
    f. I need help with toileting
    g. I choose not to answer this question

13. Do you use any of the following mobility items? (check all that apply)
    a. Hospital bed
    b. Hospital chair
    c. Cane
    d. Walker
    e. Wheelchair
    f. Toilet chair
    g. Tub or shower chair
    h. Car transfer device
    i. I do not use any of these items
    j. I choose not to answer this question

[Questions 14-17 from PROMIS-29]

14. Are you able to do chores such as vacuuming or yard work?
    a. Without any difficulty
    b. With a little difficulty
    c. With some difficulty
    d. With much difficulty
    e. Unable to do

15. Are you able to go up and down stairs at a normal pace?
    a. Without any difficulty
    b. With a little difficulty
    c. With some difficulty
    d. With much difficulty
    e. Unable to do

16. Are you able to go for a walk of at least 15 minutes?
    a. Without any difficulty
    b. With a little difficulty
    c. With some difficulty
    d. With much difficulty
    e. Unable to do

17. Are you able to run errands and shop?

a. Without any difficulty
b. With a little difficulty
c. With some difficulty
d. With much difficulty
e. Unable to do

[Questions 18-19 adapted from Cognition Self-Assessment Rating Scale (C-SARS)]

18. Do you have difficulty remembering things that happened or what people have told you?

a. Rarely
b. Sometimes
c. Often
d. All the time

19. Do you have difficulty planning ahead, making appointments, or making decisions?

a. Rarely
b. Sometimes
c. Often
d. All the time

Adapted from BRIEF Health Literacy Screening Tool

20. How often do you have a problem understanding the cause of your kidney disease?

a. Always
b. Often
c. Sometimes
d. Occasionally
e. Never

21. How often do you have a problem understanding the options for managing your kidney disease?

a. Always
b. Often
c. Sometimes
d. Occasionally
e. Never

22. How often do you have difficulty making decisions about your kidney treatment?

a. Always
b. Often
c. Sometimes
d. Occasionally
e. Never

References:

1. National Association of Community Health Centers, Inc., Association of Asian Pacific Community Health Organizations, and Oregon Primary Care Association. The Prapare Screening Tool. PRAPARE. January 6, 2023. Accessed July 1, 2023. https://prapare.org/the-prapare-screening-tool/.

2. Guide for the Uniform Data Set for Medical Rehabilitation (Adult FIM™Instrument), Version 5.1. Buffalo, State University of New York at Buffalo, 1997

3. Hays, R. D., Spritzer, K. L., Schalet, B. D., & Cella, D. (2018). PROMIS®-29 v2. 0 profile physical and mental health summary scores. Quality of life Research, 27, 1885-1891.

4. Nasrallah, Henry A. "The Cognition Self-Assessment Rating Scale for patients with schizophrenia." Current Psychiatry 22.3 (2023): 30-34.

5. Haun, J., Luther, S., Dodd, V., & Donaldson, P. (2012). Measurement variation across health literacy assessments: implications for assessment selection in research and practice. J Health Commu,17 Suppl 3:141-59. doi: 10.1080/10810730.2012.712615.

6. Morris, N.S., MacLean, C.D., Chew, L.D. et al. The Single Item Literacy Screener: Evaluation of a brief instrument to identify limited reading ability. BMC Fam Pract 7, 21 (2006). https://doi.org/10.1186/1471-2296-7-21
